# Efficient synchronization of *Plasmodium knowlesi* in vitro cultures using guanidine hydrochloride

**DOI:** 10.1186/s12936-019-2783-1

**Published:** 2019-04-25

**Authors:** Sutharinee Ngernna, Anongruk Chim-ong, Wanlapa Roobsoong, Jetsumon Sattabongkot, Liwang Cui, Wang Nguitragool

**Affiliations:** 1 Department of Molecular Tropical Medicine, Faculty of Tropical Medicine, Mahidol University, 420/6 Ratchawithi Road, Ratchathewi, Bangkok, 10400 Thailand; 2 Mahidol Vivax Research Unit, Faculty of Tropical Medicine, Mahidol University, 420/6 Ratchawithi Road, Ratchathewi, Bangkok, 10400 Thailand; 3 Department of Internal Medicine, Morsani College of Medicine, University of South Florida, Tampa, FL USA

**Keywords:** *Plasmodium knowlesi*, Synchronization, Culture, Ring, Sorbitol, Guanidine hydrochloride, Enrichment

## Abstract

**Background:**

Long-term in vitro culture of blood stage *Plasmodium* parasites invariably leads to asynchronous parasite development. The most often used technique to synchronize *Plasmodium falciparum* culture is sorbitol treatment, which differentially induces osmotic lysis of trophozoite- and schizont-infected red blood cells due to presence of the new permeation pathways in the membranes of these cells. However, sorbitol treatment does not work well when used to synchronize the culture-adapted *Plasmodium knowlesi* A1-H.1 line.

**Methods:**

A number of common solutes were tested in lieu of sorbitol for synchronization of *P. knowlesi* A1-H.1 ring stage.

**Results:**

Guanidine hydrochloride was found to selectively lyse trophozoite- and schizont-infected red blood cells, yielding highly synchronous and viable rings.

**Conclusions:**

A method for synchronization of *P. knowlesi* in human red blood cells was developed. Requiring only common laboratory reagents, this method is simple and should be applicable to most laboratory settings.

## Background

*Plasmodium knowlesi* is a simian parasite that can also cause malaria in humans [1, 2]. Whereas this parasite is prevalent in the Malaysian Borneo, its geographical range extends into the mainland Southeast Asia, Indonesia, and the Philippines [3]. Recently, the incidence of human infection of *P. knowlesi* in Malaysia and southern Thailand appears to be on the rise, despite the overall decline of malaria [3, 4]. The parasite is thus an emerging public health threat in affected areas.

Adaptation of *P. knowlesi* to continuous in vitro culture in human RBCs has opened doors to new possibilities to study this parasite [5, 6]. As common for long-term *Plasmodium* culture, *P. knowlesi* culture loses synchronicity over time. Freshly thawed *P. knowlesi* culture becomes mixed stages within 4 to 5 days. However, a synchronized parasite culture is often needed in research, particularly when the aim is to examine stage-specific phenotypes, transcriptomes, and proteomes. Several synchronization methods developed for in vitro culture of *Plasmodium falciparum* have the potential for synchronization of in vitro *P. knowlesi* culture. These methods include magnetic separation to obtain mature trophozoites and schizonts [7, 8], selective lysis of trophozoites and schizonts to obtain rings [9], physical separation based on differential density [10] or sedimentation [11], and cold treatment to obtain rings [12, 13]. Indeed, density gradient centrifugation and magnetic separation have been used successfully to obtain tightly synchronized *P. knowlesi* culture [5, 6]. However, these methods are either time-consuming or require special equipment. For *P. falciparum*, lysis of trophozoite- and schizont-infected red blood cells (RBCs), first established in 1979, remains one of the most commonly used methods due to its high efficiency, simplicity and low cost [9]. This approach exploits the increased permeability of the infected RBCs due to the new permeation pathways (NPPs) to kill trophozoites and schizonts. NPPs allow sorbitol to enter the host cell, causing influx of water which leads to cell lysis. Because NPPs are active only in the host membrane at the trophozoite and schizont stages [14], the ring-infected RBCs are resistant to sorbitol treatment.

While the sorbitol method works well to synchronize *P. falciparum*, its application to the recently human-erythrocyte adapted line of *P. knowlesi* (A1-H.1) had limited success [15]. In this study, alternative solutes were explored and guanidine hydrochloride (GuHCl) was found to selectively lyse trophozoite- and schizont-infected human RBCs to achieve synchronization of *P. knowlesi* culture.

## Methods

### *Plasmodium knowlesi* in vitro culture

The *P. knowlesi* strain A1-H.1 used in this study was from Dr. Robert W. Moon, Division of Parasitology, Medical Research Council National Institute for Medical Research, London. The parasite was cultured in complete medium (pH 7.4) containing RPMI-1640 (Invitrogen), 5.96 g/L HEPES, 2.3 g/L sodium bicarbonate, 2 g/L d-glucose, 0.292 g/L l-glutamine, 0.05 g/L hypoxanthine, 5 g/L Albumax II (Invitrogen), 0.025 g/L gentamycin sulfate, and 10% (vol/vol) horse serum (Invitrogen). Human red blood cells (RBCs) were obtained from the Thai Red Cross at 50% haematocrit and washed with RPMI-1640 containing 2 g/L sodium bicarbonate, 5.94 g/L HEPES, 2 g/L d-glucose, and 0.025 g/L gentamycin sulfate. RBCs were added to a 2–5% haematocrit and the culture was maintained in 75 cm^2^ flasks at 37 °C with gas mixture (90% N_2_, 5% CO_2_, and 5% O_2_).

### Initial testing of different solutes for synchronization of ring-stage parasites

Four solutes were tested to synchronize A1-H.1 parasite to the ring stage: sorbitol, GuHCl, glucose, and glycine. Phosphate-buffered saline (PBS) pH 7.4 was used as the control. For each test solute, a solution was prepared containing 280 mM sorbitol, 140 mM GuHCl, 280 mM glucose, or 280 mM glycine with 20 mM HEPES, pH 7.4. All solutions were filter sterilized with 0.2 μm membrane filters. The procedure was carried out at the room temperature (25–27 °C). Asynchronous parasite culture was first harvested by centrifugation at 600×*g* for 5 min. The packed RBCs (0.25 mL) was resuspended in 10 mL of each test solution by gently mixing with a pipette and incubated for 20 min. Cells were then centrifuged (600×*g* for 5 min) and the supernatant was removed. The cell pellet was washed twice with 10 mL of RPMI-1640 incomplete medium before placing into culture. For thin blood smear preparation, the pellet was adjusted to 40–50% haematocrit with complete medium. The blood smears were prepared, fixed with methanol, and stained with 10% Giemsa. The percent parasitaemia of each stage was determined under the microscope by counting intact infected RBCs from at least of 3000 cells three times.

### Final GuHCl synchronization protocol for *Plasmodium knowlesi* in vitro culture

1. Harvest cells by centrifugation at 600×*g* for 5 min at room temperature.
2. Resuspend cell pellet in at least 20X volume of 140 mM GuHCl, 20 mM HEPES, pH 7.4 and incubate at room temperature for 20 min.
3. Collect cells by centrifugation at 600×*g* for 5 min at room temperature and remove supernatant.
4. Wash cell pellet twice with at least 20X volume of RPMI-1640 incomplete medium.
5. Resuspend cell pellet in complete medium and return cells to culture under mixed gas.

### Measuring parasite multiplication after GuHCl treatment

To compare the multiplication rates of ring-stage parasites after GuHCl treatment to those obtained by density gradient centrifugation, schizonts were first purified by centrifugation of parasite culture through 55% Nycodenz [6]. After two washes with RPMI-1640, the parasites were placed back in culture with uninfected RBCs at 2% haematocrit for 6 h to allow maturation and invasion. The parasites (0–6 h old rings + remaining schizonts) were then subjected to a) GuHCl treatment or b) a second round of centrifugation through 55% Nycodenz (the bottom fraction under the Nycodenz cushion now contained only rings). Rings were then harvested, washed twice with RPMI-1640, and placed back in culture. Giemsa-stained thin smears were prepared for parasitaemia determination immediately after ring selection (0 h) and during the next cycle (36 h). Percent parasitaemia was determined by counting at least 2500 RBCs, the 95% confidence interval of which calculated according to the binomial distribution.

## Results

**Fig. 1:**
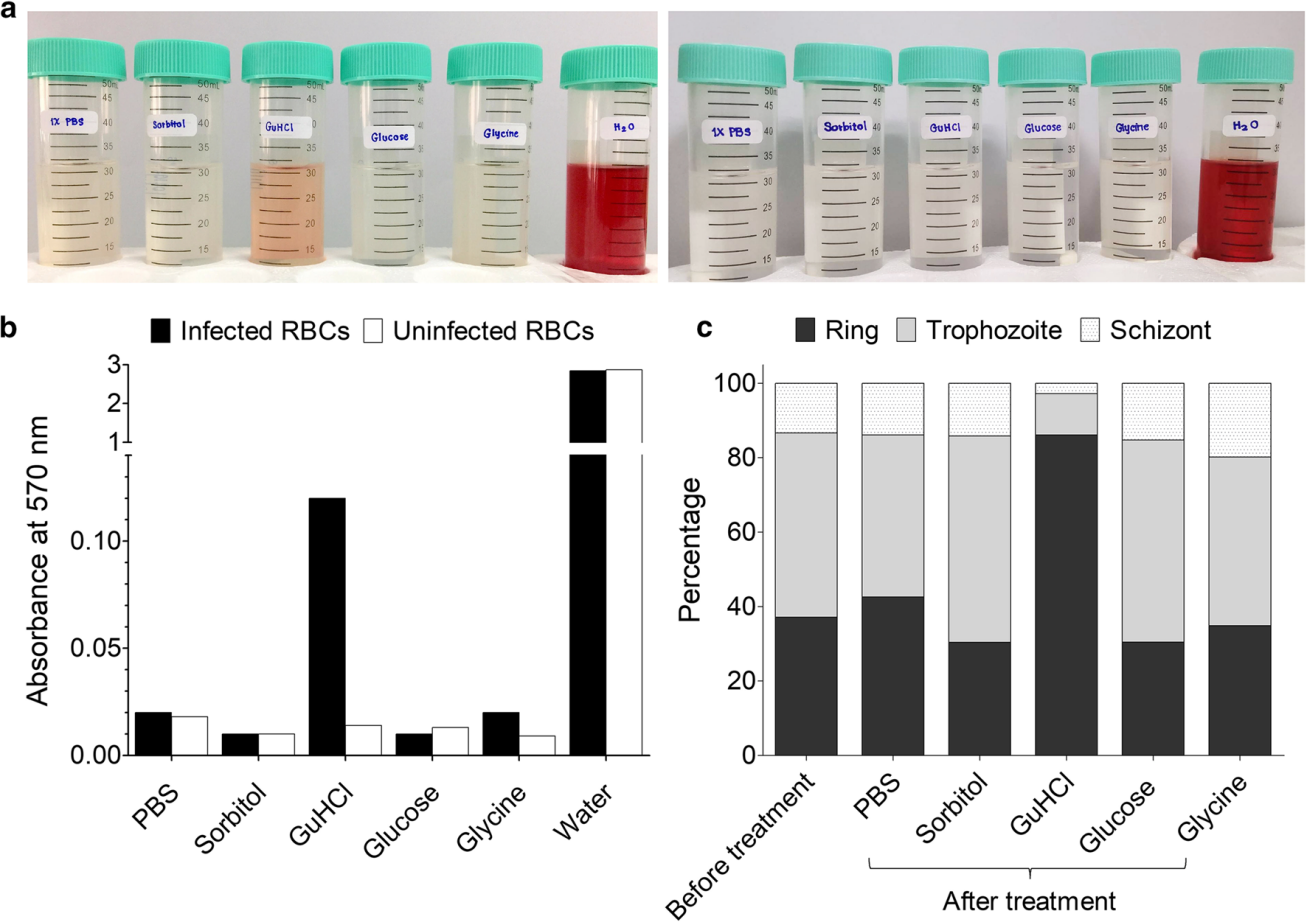
GuHCl induces lysis of trophozoite- and schizont-infected erythrocyte. **a** Left, supernatants of *P. knowlesi*-infected (1.1% ring, 1.5% trophozoite, 0.4% schizont) human RBC culture after treatment with PBS, sorbitol, GuHCl, glucose, glycine, or distilled water. Right, supernatant of uninfected RBCs after the same treatments. **b** Haemoglobin released, quantified by absorbance at 570 nm. **c** Stage distribution of the parasite before and after each treatment. Data from a single experiment in **a**

**Fig. 2:**
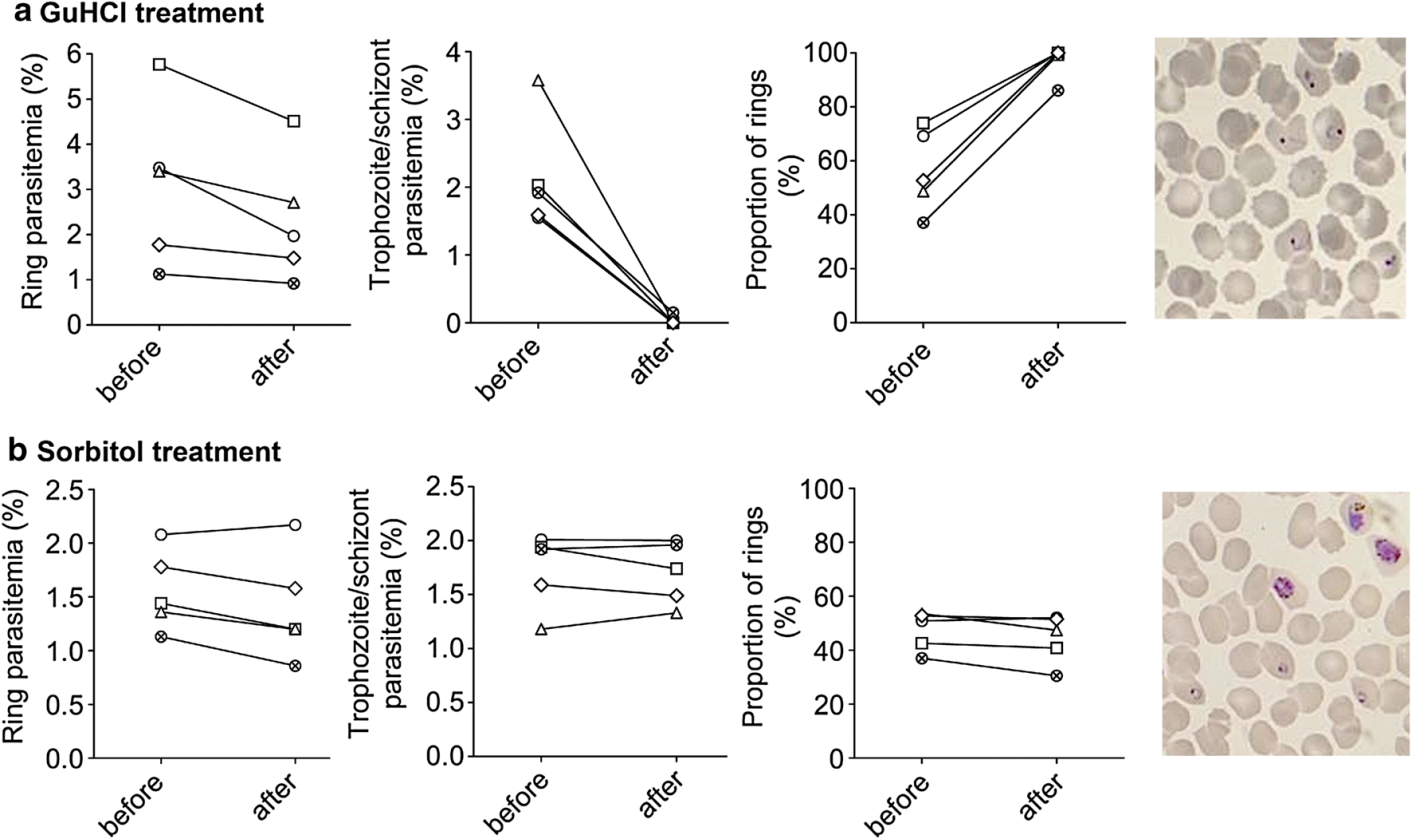
Synchronization of *P. knowlesi* A1-H.1 parasite by GuHCl. **a** GuHCl treatment. **b** Sorbitol treatment. Left to right: ring parasitaemia before and after treatment, trophozoite and schizont parasitaemia before and after treatment, proportion of rings before and after treatment, representative Giemsa-stained thin smear of infected RBC culture after treatment. Different symbols represent different biological replicates (N = 5)

To identify small molecules that could be used to synchronize *P. knowlesi* culture, a few common solutes were tested for their ability to selectively induce osmotic lysis of mature parasite-infected RBCs (Fig. 1). Asynchronous *P. knowlesi* in vitro culture was incubated in HEPES-buffered solutions of sorbitol (280 mM), GuHCl (140 mM), glucose (280 mM), or glycine (280 mM) for 20 min at room temperature. These concentrations were chosen to keep the buffers approximately iso-osmotic. PBS and distilled water were included as negative and positive controls, respectively. Treatment with sorbitol, glucose, or glycine did not cause lysis of RBCs infected with *P. knowlesi* A1-H.1, whereas GuHCl treatment resulted in lysis of infected RBCs (Fig. 1a, b). Giemsa-stained blood smears clearly showed presence of all parasite stages of the asynchronous culture after sorbitol treatment, whereas in GuHCl-treated culture the majority of remaining parasites were at the ring stage (Fig. 2). Quantification of parasite developmental stages demonstrated that GuHCl treatment disproportionally reduced the numbers trophozoite and schizonts (Figs. 1c, 2). Thus far, we could consistently obtain over 85% ring stage from a mixed culture.

**Fig. 3:**
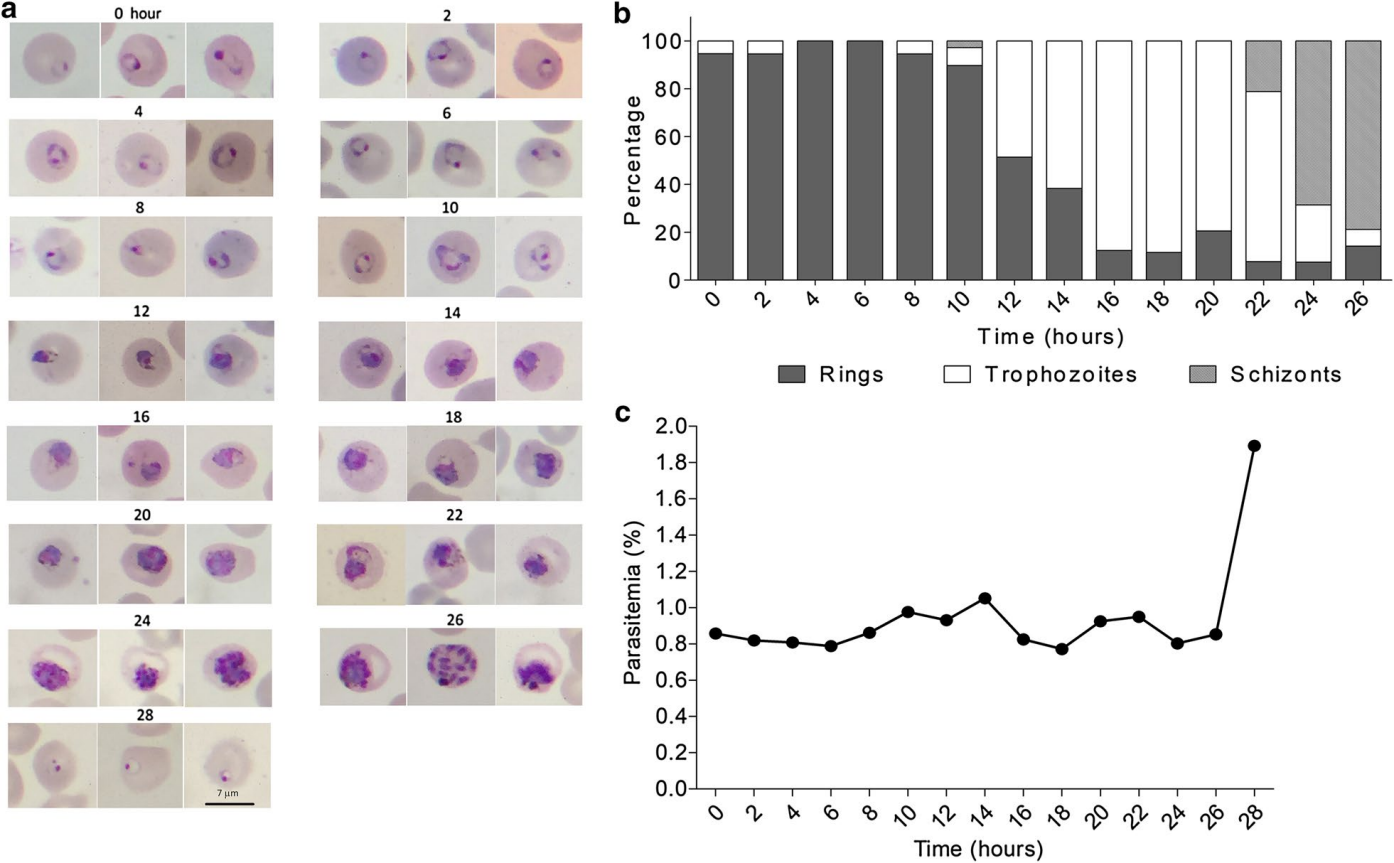
Development of GuHCl synchronized *P. knowlesi* A1-H.1. **a** Giemsa-stained thin blood smears showing the morphology of the parasites during asexual growth after GuHCl treatment. **b** Parasite stage distribution from the same experiment. **c** Total parasitaemia every 2 h after synchronization from the same experiment

**Fig. 4:**
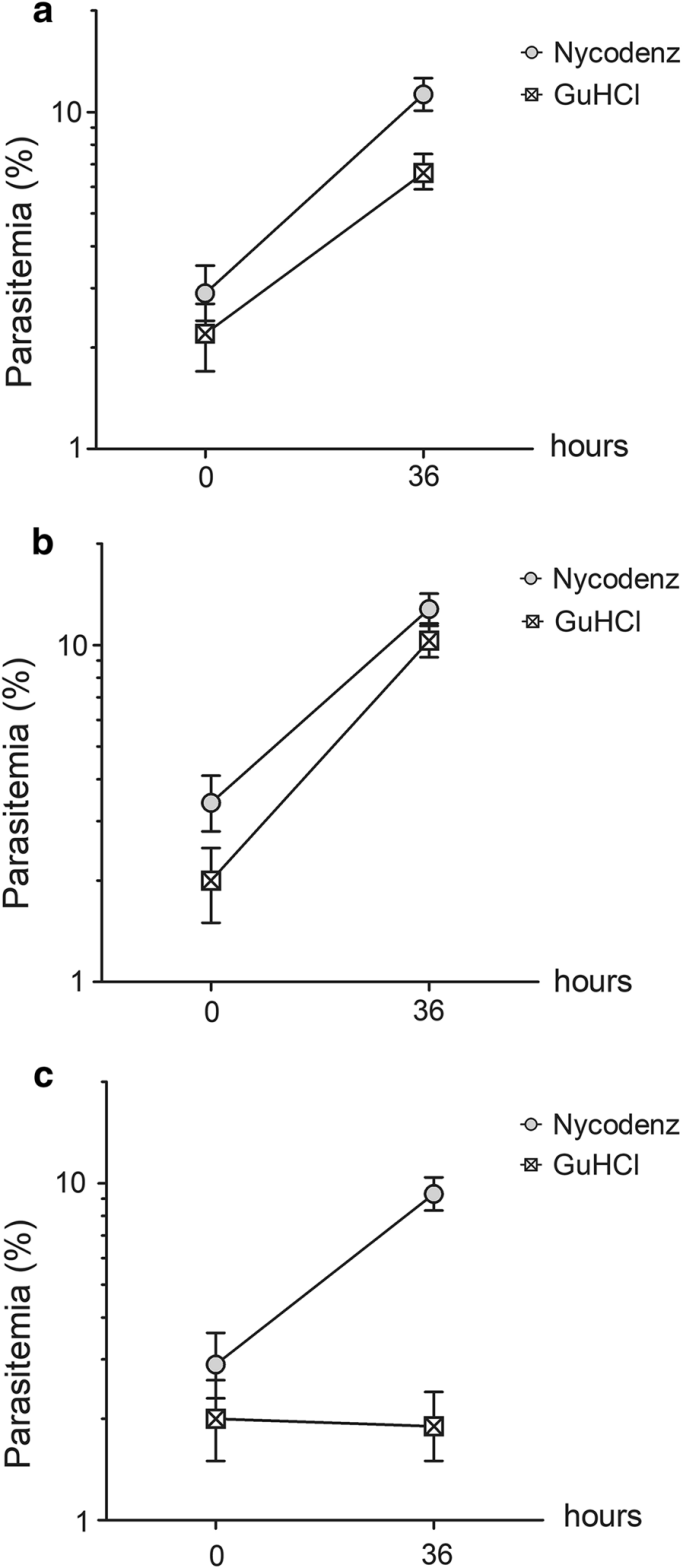
Parasite multiplication following ring synchronization by GuHCl or density gradient (Nycodenz) centrifugation. Parasitaemia was determined immediately after synchronization and at 36 h later. **a**–**c** Represent different biological replicates (A1-H.1 cultures in different human RBCs). Error bars indicate 95% confidence intervals

To test whether GuHCl treatment is toxic to the parasites, we followed parasite development after treatment. Microscopic examination of the GuHCl-synchronized ring culture every 2 h over 28 h revealed that the parasites developed normally and completed the intraerythrocytic cycle within 28 h (Fig. 3) as previously reported [6]. The multiplication rate of GuHCl synchronized parasites was also compared to that of rings purified by density gradient centrifugation (Fig. 4). In two biological replicates out of three, the fold increases in parasitaemia of these two cell preparations were similar (Fig. 4a, b), confirming normal parasite development after GuHCl synchronization. However, in one replicate (Fig. 4c), parasitaemia did not increase after GuHCl treatment. The reason for this is still unclear, but this finding suggests that GuHCl may be toxic to the parasite under some circumstance.

## Discussion

In this study, a method for obtaining a highly synchronous ring-stage culture of *P. knowlesi* is reported. The method is a modification of the standard sorbitol synchronization protocol of *P. falciparum* which does not work with the human adapted A1-H.1 line of *P. knowlesi* [15]. It was found that replacing sorbitol with GuHCl can achieve synchronization of this parasite.

The resistance of A1-H.1 to sorbitol is surprising for two reasons. Firstly, aside from a few purposefully selected *P. falciparum* laboratory lines [16–18], NPPs are considered conserved across *Plasmodium* species [14, 19–21]. Secondly, rhesus RBCs infected with *P. knowlesi* from in vitro culture are sensitive to sorbitol treatment [22]. The low sorbitol permeability of the A1-H.1 strain in human RBCs observed here may be due to mutations or epigenetic changes that occurred during the parasite’s adaptation to in vitro culture in human RBCs. The prime candidates for this permeability change are the RhopH complex components whose role was previously implicated in the NPPs activity [23–25]. Alternatively, a specific human RBC protein(s) may influence NPPs function. A comparison of the NPPs properties of A1-H.1 infected rhesus and human RBCs should be able to shed light on this divergence.

Several techniques are available to enrich the *Plasmodium* parasites at a specific stage. Most of them were developed for *P. falciparum*, including physical separation based on differential density [10], temperature cycling [26], cell cycle inhibitors [27], magnetic column purification [7, 8, 28], and Plasmion [11, 29]. For *P. knowlesi*, density centrifugation and magnetic purification have been used to isolate mature stages [6, 30, 31]. Besides these methods, the recent report of merozoite invasion inhibition by heparin and sulfated polymers suggests that these inhibitors may also be a viable alternative for parasite synchronization [32]. The method of GuHCl synchronization here offers another means to obtain the ring stage. The advantage of this method is that it is simple, fast, and scalable.

Besides *P. knowlesi* A1-H.1, there are other lines of *P. knowlesi* that have been adapted for long-term in vitro culture. These include the original H strain adapted to grow in rhesus RBCs [33] and the H_hu_ line adapted to grow in human RBCs [5]. In this study, the GuHCl synchronization method was tested only with A1-H.1 grown in human RBCs. How well the method performs with the H and H_hu_ lines is currently not known, nor is the effectiveness of the method for the A1-H.1 line grown in simian RBCs. However, because high guanidinium permeability of infected RBCs has been observed even with distantly related *P. falciparum* [34], it is possible that the GuHCl synchronization method will work universally well with other *P. knowlesi* lines as well as with *P. falciparum*.

### Limitations

GuHCl synchronization is routinely used in the authors’ laboratory. While this method works well and yields healthy ring-stage parasites most of the time, it can sometimes result in parasites with a slow multiplication rate, a risk revealed in Fig. 4c. The reason for this phenomenon is still unclear, but it may limit the utility of this method when delayed parasite growth needs to be avoided. Normal multiplication rates usually resume within one or two cycles.

## Conclusions

This study demonstrates the utility of GuHCl as an effective means for synchronization of the ring-stage *P. knowlesi* A1-H.1 in vitro culture in human RBCs. The failure of sorbitol to synchronize this line of parasite suggests that NPPs of this parasite line has a distinct solute permeability profile when compared to that of *P. falciparum*. The methodology described could be applied to identify a solute appropriate for osmotic synchronization of other parasite or parasite lines that may be resistant to sorbitol or guanidine treatment.

## Abbreviations

GuHCl: guanidine hydrochloride
h: hour
HEPES: (4-(2-hydroxyethyl)-1-piperazineethanesulfonic acid
min: minute
NPPs: new permeation pathways
PBS: phosphate-buffered saline
RBCs: red blood cells
